# Foodborne Parasites and Their Complex Life Cycles Challenging Food Safety in Different Food Chains

**DOI:** 10.3390/foods12010142

**Published:** 2022-12-27

**Authors:** Sarah Gabriël, Pierre Dorny, Ganna Saelens, Veronique Dermauw

**Affiliations:** 1 Department of Translational Physiology, Infectiology and Public Health, Faculty of Veterinary Medicine, Ghent University, 9820 Merelbeke, Belgium; 2 Department of Biomedical Sciences, Institute of Tropical Medicine, 2000 Antwerp, Belgium

**Keywords:** foodborne parasites, food chain, food safety, diagnostics, control, prevention, infection risk, meat-borne parasites, fish-borne parasites

## Abstract

Zoonotic foodborne parasites often represent complex, multi host life cycles with parasite stages in the hosts, but also in the environment. This manuscript aims to provide an overview of important zoonotic foodborne parasites, with a focus on the different food chains in which parasite stages may occur. We have chosen some examples of meat-borne parasites occurring in livestock (*Taenia* spp., *Trichinella* spp. and *Toxoplasma gondii*), as well as *Fasciola* spp., an example of a zoonotic parasite of livestock, but transmitted to humans via contaminated vegetables or water, covering the *‘farm to fork’* food chain; and meat-borne parasites occurring in wildlife (*Trichinella* spp., *Toxoplasma gondii*), covering the ‘*forest to fork*’ food chain. Moreover, fish-borne parasites (*Clonorchis* spp., *Opisthorchis* spp. and Anisakidae) covering the ‘*pond/ocean/freshwater to fork*’ food chain are reviewed. The increased popularity of consumption of raw and ready-to-eat meat, fish and vegetables may pose a risk for consumers, since most post-harvest processing measures do not always guarantee the complete removal of parasite stages or their effective inactivation. We also highlight the impact of increasing contact between wildlife, livestock and humans on food safety. Risk based approaches, and diagnostics and control/prevention tackled from an integrated, multipathogen and multidisciplinary point of view should be considered as well.

## 1. Introduction

Foodborne parasites (FBPs) have been long neglected, yet are slowly obtaining more attention, as diagnostic tools are improved and increasingly used, and burden data are becoming slowly available. Efforts made by the Food and Agriculture Organisation and the World Health Organisation (FAO/WHO) into the development of a multicriteria-based ranking for risk management of foodborne parasites have further placed the FBPs in the picture. This ranking was based on a number of criteria including amongst other number of global illnesses, morbidity, mortality; leading to a top four list related to the parasite’s public health impact: *Taenia solium*, *Echinococcus granulosus*, *Echinococcus multilocularis, Toxoplasma gondii*; and a top four of *Trichinella spiralis*, *T. solium*, *Taenia saginata*, Anisakidae when assessing their trade impact [1]. In the WHO’s burden assessment of foodborne pathogens initiative, conducted by the foodborne disease burden epidemiology reference group, the lack of knowledge of the burden of FBPs was acknowledged as well. The report lists the Disability Adjusted Life Years (DALYs) caused by 31 foodborne pathogens, including 14 parasites for which a total of 7,195,014 DALYs, 90,391,678 illnesses and 51,468 deaths were estimated for 2010. The estimates were judged conservative as often data were missing [2]. Foodborne parasites are notorious for their underreporting, most of them not having an obligatory notification.

On the level of the European Union (EU), several COST Actions including CYSTINET (TD1302) and EURO-FBP (FA1408) established or enforced Networks which have contributed greatly to the knowledge and management of FBPs in the EU.

While FBPs were previously majorly linked to endemic areas in the global south, this picture is rapidly changing due to globalisation, including increased international transports, distributions of food stuffs/products, increased trade, changing culinary behaviours moving towards less cooked, raw dishes and increased travel and migration of people. This, combined with an increase in susceptibility of a higher proportion of the global population due to, for example, a higher proportion of elderly people, leads to an increased number of people at higher risk for foodborne parasitic infections [3]. With improvements in diagnostic tools, increase in research efforts, knowledge and awareness on the impact of FBPs has increased, yet a lot of gaps are remaining.

A challenge typically related to FBPs is their long incubation times in humans, whereby clinical symptoms may take even years to appear (e.g., cyst stages of *Echinococcus* spp., *T. solium*) which complicates diagnosis, as well as the establishment of the original source of infection.

The FBPs often represent complex, multi host life cycles with parasite stages in the hosts, but also in the environment, where parasite stages may survive for many months or even years. Parasite stages may also contaminate other food stuffs such as fruits and vegetables. A human infection risk therefore may occur (even for a single parasite) at several points in different food chains.

Reduction of the risk can and should be envisaged at different points as well, following a One Health approach, accompanied by an efficient detection (and monitoring) of infection in different hosts. Yet, as will be exemplified below, currently, on a global level, many FBPs are not or insufficiently controlled, diagnostic tools not used or characterised by an insufficient performance. Furthermore, generally, levels of awareness by the different stakeholders are still too low.

**Figure 1:**
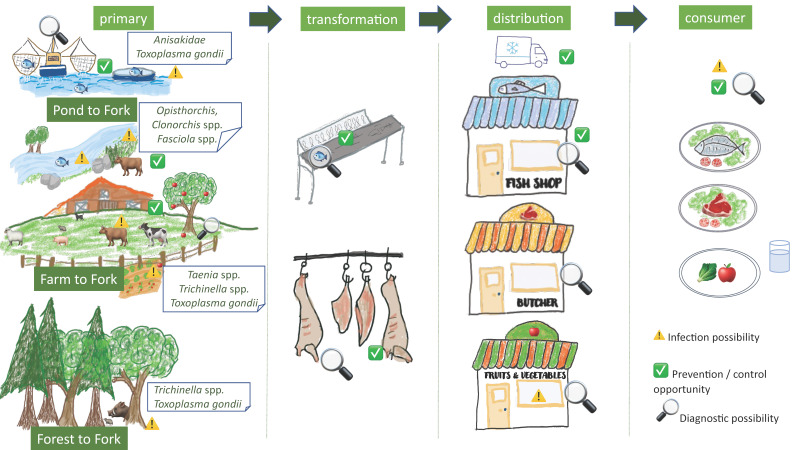
Simplified presentation of the foodborne zoonotic parasites in the different food chains, and identification of sites for diagnosis and control/prevention.

In this Special Issue on safety of the food chain, this manuscript aims to provide an overview of a number of important zoonotic foodborne parasites, with a focus on the different food chains in which parasite stages may occur. This paper focusses on the human infection risk, on safety of the food chain, and as such does not cover diagnosis/treatment in human patients. We have chosen some examples of meat-borne parasites occurring in livestock (*Taenia* spp., *Trichinella* spp. and *Toxoplasma gondii*), as well as *Fasciola* spp., an example of a zoonotic parasite of livestock, but transmitted to humans via contaminated vegetables or water, covering the ‘*farm to fork*’ food chain as well as meat-borne parasites occurring in wildlife (*Trichinella* spp., *Toxoplasma gondii*), covering the ‘*forest to fork*’ food chain. Moreover, fish-borne parasites (*Clonorchis* spp., *Opisthorchis* spp. and Anisakidae) covering the ‘*pond/ocean/freshwater to fork*’ food chain were added (Figure 1).

## 2. Farm to Fork and Forest to Fork

In this chapter, four important meat-borne zoonotic parasites from livestock will be discussed, including *T. solium*, *T. saginata*, *Trichinella* spp. and *Toxoplasma gondii*, as well as *Fasciola* spp. in the framework of the *Farm to Fork food chain*, while for *Trichinella* spp. and *Toxoplasma gondii* the *Forest to Fork food chain* will be highlighted as well (Figure 1).

### 2.1. Taenia solium, Taenia saginata

#### 2.1.1. Introduction

*Taenia solium* and *T. saginata* are two human tapeworms. *Taenia saginata* has cattle as the intermediate hosts and primarily represents an economic burden to the meat sector. *Taenia solium* has pigs as intermediate hosts, but also humans may act as accidental intermediate host, developing neurocysticercosis, a main cause of epilepsy in endemic areas. *Taenia solium* therefore represents not only an economic problem in the human and veterinary sector, but also a serious public health problem. *Taenia saginata* has a global distribution, occurring in a high number of countries, with higher prevalences described in countries with raw meat consumption practices such as Belgium [4,5].

After consumption of undercooked infected pork/beef, a tapeworm develops in the human intestine (taeniosis). Eggs are shed from the gravid proglottids, which leave the host actively (only for *T. saginata*) or with the stool and subsequently contaminate the environment. Taeniosis generally leads to no/limited clinical signs and symptoms, though abdominal complaints, nausea and weight loss have been reported [4].

The metacestode larvae (cysticerci) develop in the (accidental) intermediate hosts after ingestion of the eggs (cysticercosis). In the natural intermediate host, the cysticerci lodge in the muscles, but also subcutaneously and in the brain (porcine cysticercosis, bovine cysticercosis). Generally speaking, the pig/cattle host shows no clinical signs or symptoms, though seizures and changes in behaviour have been described in heavily infected pigs [6,7]. For cattle, heavily infected carcasses detected at slaughter are condemned, while lightly infected carcasses require treatment, but can be sold afterwards. Nevertheless, the freezing of carcasses also entails a value loss. In Belgium, farmers pay an insurance to cover their losses due to bovine cysticercosis, the cattle owners bear an economic cost estimated at €3,408,455/year, while on the human side, an economic cost of €795,858/year was estimated for the taeniosis cases [8]. For porcine cysticercosis, infected carcasses should be condemned at slaughter, leading to economic losses for the farmer or trader. While meat inspection is often not implemented in rural areas in the Global South where most infections occur and therefore carcasses not condemned, infected carcasses do frequently represent an economic loss, as the value of the carcasses may be reduced by 50% [9]. For example, in Tanzania, a loss of nearly three million USD was estimated due to porcine cysticercosis in 2012 [10]. In the human accidental intermediate host, *T. solium* cysticerci may also lodge in the muscles, subcutaneously, and in the central nervous system. The latter is responsible for most clinical signs and symptoms including seizures, epilepsy, severe progressive chronic headache, vision problems etc. [4]. *Taenia solium* has been recognised as the FBP with the highest burden, estimated conservatively at 2,788,426 DALYs [2].

#### 2.1.2. Localisation of the Infection Risks for the Consumer in the Food Chain

People develop taeniosis after consumption of undercooked infected pork/beef with viable cysticerci. When the infected pork/beef reaches the consumer in the food chain, the risk of exposure depends on the culinary practices of the consumer (see Section 2.1.4).

For *T. solium*, causing human cysticercosis, the routes of human infection are more complex, multi causal, and do not require the pig host as the transmission is human-to-human either direct or indirect. Eggs excreted by a human tapeworm carrier are immediately infective. Eggs may be ingested by tapeworm carriers or by close contacts of the carrier via the fecal oral route due to insufficient hand hygiene [4]. Eggs may also end up in the environment and contaminate fruits, vegetables, soil, water and surfaces [11], all potential sources of human infection. In areas with poor sanitation this happens in a more direct way via open defecation, equally so human stool may be used as a fertiliser for vegetable gardens. On the other hand, *Taenia* spp. eggs have been detected in sewage, in water purification plants, including effluent of these plants, indicating an insufficient clearing of the eggs from the dirty water entering the plants [11,12]. The role of insects in the dispersal of eggs in the environment needs clarification, and their importance for transmission of infective eggs to the human host is unknown [12]. The importance of the different routes of transmission to humans, either direct via contact with a carrier, or indirect via fruits, vegetables, water, etc., needs further research.

Also the survival time of the eggs in different environmental matrices (e.g., soil, water, …) under different climatic conditions, and as such their potential infectivity to humans/animals needs further investigation. Studies have been conducted, though primarily for *T. saginata*, indicating timespans of survival of up to one year and also the capability of the eggs to survive a European winter [13] (reviewed by [12]).

#### 2.1.3. Diagnostic Options in the Food Chain

In the food chain, different points of diagnosis are possible. For taeniosis, infective cysticerci (viable) should not enter the food chain, therefore, infected carcasses should be picked up and removed from the food chain or should be treated (for *T. saginata*) [4]. There are two major hurdles in this detection, (1) the implementation of meat inspection and (2) the sensitivity of meat inspection. While meat inspection is implemented on most/all carcasses in industrialised countries, this is not the case in a lot of countries in the Global South, where backyard slaughter, or slaughter in illegal slaughterslabs is routine practice. Even though there seems to be an increasing level of knowledge and awareness regarding the risk of cysticercosis [14], the knowledge is still limited, and the risk perception insufficiently high. Farmers/butchers will still allow the infected meat to reach consumers, often for economic reasons [15]. As an alternative, farmers and traders may conduct a tongue inspection on the live pig, before purchasing. While this system works reasonably well in heavily infected pigs, the sensitivity is very low for other levels of infection, allowing a high number of infected carcasses to enter the food chain [16,17].

Meat inspection has a high specificity, but a notoriously low sensitivity, especially in lightly infected carcasses. For porcine cysticercosis, a sensitivity of 22% has been determined [16], for bovine cysticercosis, this has been estimated at 0.54% in Belgium, where primarily low levels of infection were observed [18].

Serological tools, detecting circulating antigens or specific antibodies have been developed, both for porcine and bovine cysticercosis. Yet, here also, though sensitivities are usually (somewhat) higher than for meat inspection, the tests have specificity problems. For circulating antigen detection, cross reactions with *Taenia hydatigena* are common, which is especially a problem for porcine cysticercosis, as in a number of endemic areas there is a co-occurrence of these parasites [16].

Most available molecular test are used for confirmation of detected lesions in the meat (reviewed by [19] for porcine cysticercosis).

Diagnostic options in the food chain related to human cysticercosis (*T. solium*) are currently not routinely implemented. Tests to detect eggs on fruits/vegetables or in environmental matrices such as water, soil have been reviewed recently by [11], with as main conclusions a general lack of sensitivity largely due to low recovery rates of the eggs from the matrices; specificity issues when only microscopic detection is included; and mostly a complete lack of standardisation of tests.

#### 2.1.4. Prevention and Control Options in the Food Chain

Detection and removal of infected carcasses from the food chain (or treatment of lightly infected carcasses) is one of the main preventive targets for taeniosis. As mentioned above, this target is complicated by the lack of implementation of meat inspection in certain highly endemic areas, and even if implemented, especially lightly infected carcasses may still enter the food chain.

For bovine cysticercosis, the impact of meat inspection has been estimated in Belgium, where a prevalence of 42.5% was determined [18]. Meat inspection would only pick up 408 viable cysticerci from an estimated total of 213,344 viable cysticerci, present in the infected carcasses. Important here is that the performance of meat inspection is particularly low in the Belgian setting as most carcasses have very light infections. Nevertheless, in the same study, implementing a serological tool, in this case the circulating antigen detecting ELISA, would in a ten-year period greatly reduce the occurrence of bovine cysticercosis from >40% to 0.6%. The European legislation does allow for serological testing (EC 2019/627 [20]), but in practice, this is not yet implemented, probably due to the costs related to this approach.

In the EU, a more risk-based meat inspection approach is currently implemented considering age of the animals and their production system allowing a selection of animals to undergo a more in-depth meat inspection [21].

As for other FBPs, production systems are of major importance in allowing parasite access the animal hosts. In highly confined and controlled systems (see Section 2.2.4) pigs/cattle would not have access to *Taenia* eggs due to controlled feed and water access, high hygiene levels avoiding direct contamination via a potential human tapeworm carrier, etc. Nevertheless, while for pigs this is the case in a lot of commercial farming systems, for cattle this is much less so, as outdoor grazing is common; and with the trend towards more biological/organic farming, (renewed) access to potentially contaminated environment/feed/water is created [3].

Vaccination of cattle and pigs has been a subject of research for many years [22]. Especially for the pig host a number of vaccines have been developed, and have been evaluated in the field. The TSOL 18 vaccine is now commercially available, and has shown a very high efficacy, in combination with a single treatment with oxfendazole [23,24]. The latter, treatment with oxfendazole, has also been proposed as an effective option for the control of porcine cysticercosis [25], yet is not implemented outside scientific studies.

At the end of the food chain, the consumer may play a major role as well. Culinary habits will greatly influence the inactivation of viable cysticerci in pork/beef. As for other FBPs, good cooking practices avoiding raw/undercooked pork/beef consumption practices play an essential role in the level of risk of exposure of the consumer, leaving an important role to health education and awareness creation [25,26].

Of course, interventions directed towards the human host such as treatment of tapeworm carriers, stopping open defecation, or management of sludge on pastures are highly relevant [25], but not the focus of this food chain-oriented manuscript.

### 2.2. Trichinella spp.

#### 2.2.1. Introduction

Larvae from the *Trichinella* genus present in different meat and meat products have been a source of infection for humans for centuries, with outbreaks still occurring regularly today, with a global distribution.

Transmission of *Trichinella* spp. among non-human animals occurs by predation or carrion consumption. Transmission to humans occurs via the consumption of raw or undercooked meat [27], whereby infective larvae situated within the muscle cells are released in the stomach to further mature in the small intestine into adult worms. Subsequently, after mating, adult females produce larvae that will migrate to the muscles. Myalgia, diarrhea, fever, facial edema and headaches were mostly reported as clinical signs and symptoms in infected people. Most of these disappeared within 2–8 weeks after treatment, nevertheless myalgia and fatigue may remain present for years. Early diagnosis and treatment in the last decades probably have contributed to a decrease in mortality due to trichinellosis [27]. *Trichinella* spp. were detected in domestic and wild animals in 66 countries and in humans in 55 countries [27,28]. Trichinellosis represents 550 DALYs, with an estimated 4470 illnesses globally in 2010 [2]. A review conducted to assess the global incidence and clinical impact of trichinellosis identified 65,818 cases from 41 countries between 1986 and 2009. Reporting of cases varies greatly though, and is mainly linked to hospitalised cases, representing a serious underestimated from the true number of cases, including mild or asymptomatic cases. The World health Organisation European Region accounted for the majority of the cases (87%) [27] occurring primarily in adults.

Two life cycles are described for *Trichinella* spp., a domestic and a sylvatic cycle. The domestic cycle, primarily involving pigs and rodents as hosts and the encapsulated species *Trichinella spiralis* (T1), relates in this manuscript to the *Farm to Fork food chain*.

The sylvatic cycle including a large range of wild mammals, birds and reptiles [29], relating to the *Forest to Fork food chain*, includes primarily the other *Trichinella* species and genotypes. The encapsulated clade with species infecting mammals only, e.g., *Trichinella nativa* (T2), *Trichinella britovi* (T3), *Trichinella murelli* (T5), *Trichinella nelsoni* (T7), *Trichinella patagoniensis* (T12), and *Trichinella chanchalensis* (T13), with three genotypes *Trichinella* T6; *Trichinella* T8 and *Trichinella* T9, and less common *T. spiralis* (T1). Species from the non-encapsulated clade infecting mammals and birds include *T. pseudospiralis* (T4) and infecting mammals and reptiles include *T. papuae* (T10) and *T. zimbabwensis* (T11) [28,29].

A recent paper by [30] reviews the presence of *Trichinella* in wildlife globally, identifying the polar bear (57.58%), martens (32.39%) as the species with the highest prevalence in the palearctic region. While most studies cover terrestrial mammals with as most studied the wild boar, red fox, raccoon dog, wolf, black and polar bears, *Trichinella* has been detected in marine mammals and birds as well. In addition, rodents and lagomorph species were found with *Trichinella*. These animal species are important for the maintenance of the transmission cycle, at the same time may be hunted for food consumption, yet are often not part of the food safety control systems. While lions and hyenas have been reported as hosts, and again play a role in the maintenance of the life cycle, infection detected in crocodiles presents a higher risk for unsafe food for humans. The zoonotic potential of *T. zimbabwensis* is still a matter of debate, an increased use of molecular tools to identify larvae either from a patient (after a muscle biopsy) or after tracing back the meat source will bring more clarity.

#### 2.2.2. Localisation of the Infection Risks for the Consumer in the Food Chain

People are infected via the consumption of infected, undercooked meat. While most of the human infections/outbreaks are associated with pork and pork products from pigs from outdoor breeding farms, cases of infection via consumption of insufficiently cooked other meat and meat products including game meat, especially from wild boars, have been reported regularly [27]. Examples of outbreaks related to horse meat in France and Italy [31,32], dog meat in China [33] have been described. Two large trichinellosis outbreaks in France with direct parasitological evidence indicating horse meat as the source of infection included 128 and 407 cases (reviewed by [31]).

Pork and pork products originating from pigs kept under highly confined and controlled conditions is not a source of infection if the conditions are properly implemented and maintained (see below). Uncontrolled pork/meat/game meat often consumed at the household level, the latter linked to hunting activities, has been regularly reported as source of infection. As described above, a high number of wildlife species may be infected with *Trichinella* spp., and may therefore be potential sources of infection. Moreover, illegal import of uncontrolled meat from endemic areas has led to outbreaks. International travellers returning home from endemic areas may also develop disease upon their return [27].

Within the limitations of the diagnostic tests, controlled pork/meat/game meat (after slaughter) should prevent infected meat reaching the consumer, or at least at infection levels leading to clinical disease.

#### 2.2.3. Diagnostic Options in the Food chain

Direct testing of muscle samples collected from pig carcasses at the slaughterhouse based on the artificial digestion method is now the most often implemented diagnostic technique, while previously, compression of a small amount of (porcine) muscle between glass slides followed by microscopic examination was routinely applied [34]. Pooled samples of pig muscles are analysed by the magnetic stirrer digestion method (in acidified pepsin). Allowing testing of multiple samples saves a substantial amount of time, especially in low prevalence situations, even though a positive batch result has to be followed by smaller batch testing. The method has a high specificity when conducted by well trained staff. As detected larvae can be recovered, subsequent molecular identification majorly helps epidemiological investigations. Lack of a high sensitivity is often mentioned as a major disadvantage, as infection levels of 3–5 larvae in one gram of muscle would be needed to obtain a sensitivity close to 100% [34]. Nevertheless, application of the test does allow detection of carcasses at this minimum infection level, which are exactly those carcasses that present the highest risk for causing clinical disease in humans [35]. As the food safety objective is to avoid clinical trichinellosis, the performance is satisfactory.

For game, hunters are advised (or obliged depending on the applying regulations) to collect muscle samples for testing.

Indirect detection of infection by specific antibody detection in serum via Enzyme Linked Immunosorbent Assays (ELISAs) is currently not advised for the determination of the infection status of individual carcasses in a food safety control system. Antibody detection suffers from insufficient sensitivity and specificity, though this has been a matter of debate [32,36]. Cross reactions may occur with other parasites infecting pigs, though the level is strongly dependant on the antigen used in the ELISA. Use of properly prepared excretory-secretory (ES) antigens, or ES products, recombinant and synthetic antigens with the same dominant epitopes have been claimed to provide a good specificity in ELISA [36]. Nevertheless, taking into consideration potential false positive results, positive serology would have to be followed by a direct test to confirm infection. The ELISA is usually characterised by a higher sensitivity than the artificial digestion, with a reported detection of one larva per gram of tissue [35,36]. However, this performance is related to the dose of infection, which will influence the level and timing of presence of detectable levels of specific antibodies in the serum, whereby low infection doses may be detectable only as early as six weeks after infection, allowing for a rather large diagnostic window of false negativity. This particularly renders the ELISA much less suitable for individual carcass assessment, as while the test result will be negative, *Trichinella* larvae may already be present in the muscles. Heavy infections (large infections dose) on the contrary, can be picked up as early as seven days post infection. However, as [35] debates, these low levels of infection may be missed by the direct detection methods as well, as these methods require a presence of 3–5 larvae per gram of tissue to be detectable. Nevertheless, given the challenges related to both the ELISA’s diagnostic specificity and sensitivity, currently the artificial digestion method is still the method of choice recommended by the scientific community and described in regulatory documents.

On the contrary, ELISA can be useful for surveillance or monitoring in pigs and other animals [35] at primary level, in live animals.

Besides the diagnostic performance of the test, cost plays an important role as well. Barlow and colleagues [37] reviewed the available tests for assessment of safe pork and estimated the artificial digestion to be the most optimal choice. The possibility for analyses of pooled samples greatly contributed to a cost reduction, allowing for a system with a satisfactory diagnostic performance and an acceptable cost. ELISA was not selected for individual carcass assessment for reasons explained above. Molecular techniques, such as the conventional and real time PCR, while ideal to identify the *Trichinella* larva(e), are currently not cost efficient for large scale testing of carcasses. Nevertheless, with technological advances, molecular based methods may become more plausible replacements of the artificial digestion [37]. A promising example could be the lateral flow- recombinase polymerase amplification (LF-RPA) targeting the mitochondrial small subunit ribosomal RNA (rrnS) gene, which can be applied to fresh and frozen pork samples. Besides a very high sensitivity, it has a reported 100% specificity, quick time to result (less than 20 min) and relatively low equipment needs [38]. Nonetheless, a more large-scale evaluation on meat samples is needed [37].

#### 2.2.4. Prevention and Control Options in the Food Chain

In the farm to fork food chain, direct systematic testing using the artificial digestion (see Section 2.2.3) of individual pig carcasses has been a cornerstone of clinical human trichinellosis prevention in many countries for many years. While testing removes a number of infected carcasses from the food chain, it does not detect the lightly infected carcasses and as such does not remove the parasite from the food chain, neither does it fully prevent human exposure to the parasite [35]. Still, the aim to avoid human clinical trichinellosis cases can be achieved implementing this system [39]. Moreover, detection of (heavily) infected carcasses and subsequent correct removal of these carcasses, avoids further potential transmission via animal feed for example. Of course, systematic testing comes with a high cost. In the European Union, this was estimated at an annual cost ranging from 25–400 million euro [40].

With the changes in farm management system, moving towards highly confined and highly controlled housing and farming systems (high levels of biosecurity), the risk of exposure of the pig host is removed and the occurrence of *Trichinella* spp. in the pig host subsequently dropped drastically. Indeed, under these conditions, the parasite may be removed from this particular farm to fork food chain. The development of international guidelines describing criteria for controlled housing and management, thereby prevented the risk of exposure of animals. Implementation of these systems actually reduces/removes the need for testing [35], as the parasite is absent from these particular commercial pork production systems. Indeed, summary data from the EU (European Centre for Disease Prevention and Control, [41]) for 2019 described that routine slaughter testing of 72.8 million pigs raised under controlled housing reported no *Trichinella* spp. infections. Considering the cost of testing, especially in low prevalence settings, with most animals testing negative, alternatives were searched to replace the systematic testing.

A set of criteria described by the World Organisation for Animal Health (OIE) to recognise free regions or countries was fairly rapidly replaced by a set of requirements to meet for a status of negligible risk as advised by the International Commission on trichinellosis [35,42]. The latter has as a purpose to ensure food safety (consumer health) and to obtain standardised requirements for international trade, removing the need for testing of animals originating from herds classified as negligible risk. The standards included amongst other good feed manufacturing and storage practices, rodent control, prevention of pigs accessing wildlife, removal of deceased pigs and controlled animal movement [35].

Of course, not all pigs are bred under these controlled management systems, and as such, are exposed to a higher risk of *Trichinella* spp. infection as exemplified by the EU summary data whereby 218 positive pigs were detected from 139.6 million pigs from non-controlled housing tested (European Centre for Disease Prevention and Control, [41]). Non-controlled housing may allow pigs access to potentially infected rodents and wildlife. The same counts for other animal species bred under non-controlled systems, such as horses. Carcasses from the latter also need to be tested systematically, like pigs. The trend towards biological/organic pig farming [3], moving away from these highly controlled/confined systems opens opportunities for the parasite to enter the farms. Whether there is a risk and how high this risk is, depends on the infection levels in the local wildlife, including rodents. In this interface area setting, both food chains, farm to fork and forest to fork merge. The expansion of wild boars into areas of free-range pig production in the Unites States represents a risk for pig infection, leading to the introduction of *Trichinella* spp. into the farm to fork food chain [43], at the same time increasing the availability of potentially infected wild boar meat via hunters.

For *Trichinella* spp., the forest to fork food chain is an important source of infection to humans, especially game meat consumers, hunters. Human behaviour is particularly important in this food chain, as the testing (or not) of game meat, and consumption practices are dependent on the hunter/consumer. Often home consumption of hunted game is not subject to regulations, therefore testing is frequently not performed. Risk of infection is subsequently dependant on the presence and level of infection as well as on the culinary practice of the household which may include cooking, freezing or curing, influencing the inactivation of the parasite. Health education on good preparation methods for meat that might contain *Trichinella* larvae, as described by the International Commission on Trichinellosis is encouraged, including cooking, freezing (for meat from domestic pigs), and irradiation [34].

Efforts have been ongoing in the development of vaccines targeting to reduce the larval or adult worm burdens, but to date, no sufficiently effective vaccine is available [44].

As highlighted in Section 2.2.3, ELISA detecting specific antibodies may be used for surveillance and monitoring in pigs and other animal species [36].

### 2.3. Toxoplasma gondii

#### 2.3.1. Introduction

*Toxoplasma gondii* is an obligate intracellular protozoon that causes toxoplasmosis in man and animals. Toxoplasmosis is one of the most common parasitic zoonoses worldwide with an estimated one third of the global population being infected [45]. Toxoplasmosis is present in every country and seropositivity rates range from less than 10% to over 90% [45]. FAO and WHO have ranked *T. gondii* fourth out of 24 foodborne parasites of global importance [1]. The global annual incidence of congenital toxoplasmosis was estimated to be 190,100 cases. This was equivalent to a burden of 1.20 million DALYs (95% CI: 0.76–1.90). High burdens were seen in South America and in some Middle Eastern and low-income countries [46].

*T. gondii* uses felines as the definitive hosts, but is less specific when it comes to the intermediate hosts, which can be any warm-blooded animal, including mammals and birds. The lifecycle comprises an intestinal sexual phase that takes place only in the final hosts and results in the production of unsporulated oocysts that are faecally shed by infected felines and sporulate in the environment; and an extra-intestinal phase in intermediate hosts which comprises the formation of tachyzoites and tissue cysts that contain bradyzoites. In most hosts, *T. gondii* causes a lifelong latent infection in tissues such as skeletal and heart muscles, visceral organs and the central nervous system [47].

Most infections with *T. gondii* in humans and animals are subclinical or cause mild clinical signs and symptoms. Severe disease may occur in hosts with an immature or compromised immune system, or in case of higher pathogenicity of the parasite strain. In livestock, *T. gondii* is an important cause of abortion in sheep and goats. In humans, infection with *T. gondii* is particularly important in pregnant women and in immunocompromised people. When primary infection occurs during pregnancy *T. gondii* can cross the placenta and reach the foetus causing a mild to life threatening infection depending on the gestational stage. Disease- or medication-induced immunosuppression can lead to encephalitis or disseminated toxoplasmosis in adults. While toxoplasmosis in immunocompetent individuals is asymptomatic in around 80% of cases, in about 20% infection it may cause fever, mononucleosis-like symptoms, or ocular manifestations such as chorioretinitis [47].

#### 2.3.2. Localisation of the Infection Risks for the Consumer in the Food Chain

Infection in humans may occur from the accidental ingestion of oocysts that can be present in food such as vegetables and fruits but also shellfish, or in water due to environmental contamination with cat faeces; or from the consumption of raw or undercooked meat containing tissue cysts. As mentioned above vertical transmission may occur when a parasite-naïve women gets infected during pregnancy. Less frequent ways of infection are caused by drinking goat milk containing tachyzoites during the acute phase of infection or by transplantation of tissues from a *T. gondii*-infected donor. Consumption of undercooked infected meat is considered a major risk factor for humans, especially in Europe, where it has been associated with 30–63% of infections [48,49]; Hill et al. [50] demonstrated the predominance of oocyst-caused infections in North America. Based on the relative proportion of the different animals in the overall meat consumption, the proportion of meat types consumed raw or undercooked, the exposure and susceptibility of different animal species to *T. gondii* and the subsequent establishment and survival of parasites in their tissues, meat from pigs and small ruminants are to be seen as the main sources of infection (farm to fork food chain). Meat from wildlife (forest to fork food chain), horses, poultry and cattle are less common sources [49].

*Toxoplasma gondii* is an example of a health issue that can be directly connected to outdoor animal husbandry [51]. Grazing ruminants mostly acquire infection by ingestion of oocysts from the environment. As a result, the seroprevalence in sheep tends to be high, e.g., between 27.8% and 87.4% in West-European countries [52]. Pigs may become infected both by ingesting oocysts and by the consumption of meat containing tissue cysts from infected rodents or kitchen leftovers. Consequently, a low prevalence (0–1%) is found in pig farms with well-managed controlled housing conditions that practise rodent control, keep cats away from the farm and the feed, and restrict the access to the farm. In contrast, a high prevalence of up to 60% is found in poorly managed or free-range farms [53,54,55]. Outdoor access of pigs considerably increases the risk for *T. gondii* infection such as in free-range organic farms [54,56]. Thomas et al. [57] studied the detailed anatomical distribution of *T. gondii* in naturally and experimentally infected lambs and found that parasite DNA could be detected in all the edible parts.

Environmental contamination with *T. gondii* oocysts is understudied and likely underestimated, which is partly due to the lack of suitable harmonized sampling approaches and detection methods. Oocysts can be spread in the soil by arthropods, earthworms, wind, and rain [58]. Sporulated oocysts are highly resistant and can remain infective in soil for up to two years [59]. Several waterborne infections associated with *T. gondii* oocysts have been described. Water in irrigation systems, rivers, lakes, beaches, and coasts, as well as wastewater and groundwater can be contaminated with the environmentally resistant oocysts. Moreover, oocysts can survive various inactivation procedures using chemical reagents, including sodium hypochlorite and chlorine [60]. Oocyst contamination of fresh vegetables may occur through cultivation in contaminated soil or using contaminated water for irrigation or washing. Consumption of raw vegetables and fruits are a risk factor. *T. gondii* oocysts can also enter the marine environment through disposal of sewage and water runoff, where they can cause infections in marine animals [61]. Oocysts have been detected in wild and commercial bivalve mollusks which are filter-feeders and can concentrate microorganisms. They can retain viable *T. gondii* oocysts for 85 days following uptake [62]. These shellfishes can pose another risk for consumers when consumed undercooked or raw [63].

#### 2.3.3. Diagnostic Options in the Food Chain

*Toxoplasma gondii* infections can be detected by direct and indirect techniques. Coprological methods are used to detect oocysts in cat faeces. (Immuno-)histological and molecular methods and bioassay are used to detect the parasite in tissues, in most cases post-mortally. Recently, an improved molecular method using magnetic capture combined with RT-PCR has been developed that allows the detection of the parasite in larger portions of tissue [64]. Cat and mouse bioassays are the reference direct techniques to isolate *T. gondii*; however, these tests are not commonly used due to the long time it takes to obtain results, ethical issues, and high costs [47]. An alternative method is cell culture which is limited in use because of the variability of the results [65]. Many serological methods (indirect detection) have been developed and validated in humans and several animal species. Among these techniques are the immunofluorescent assay (IFAT), enzyme-linked immunosorbent assay (ELISA), latex agglutination tests (LAT) and the modified agglutination test (MAT). An important question is whether seropositivity can be linked to the infectivity of tissues. Opsteegh et al. [66] found that the correlation between antibody detection against *T. gondii* and direct parasite detection is high in pigs, small ruminants, and chickens. In these species, the use of serology can help determine the risk to the consumer, but it may not be as useful in other species, such as horses and cattle. In addition, a seronegative result does not necessarily mean that the meat is free of *T. gondii* [67].

#### 2.3.4. Prevention and Control Options in the Food Chain

Although *T. gondii* is a high priority foodborne zoonotic parasite, it is not systematically controlled. At present, there are no specific regulations and no standardised methods for the detection of *T. gondii* in any food matrix. Because chronically infected animals are mostly asymptomatic and tissue cysts cannot be detected during routine meat inspection—the size of tissue cysts is less than 100 µm—most infected carcasses pass meat inspection and enter the food chain.

Currently, most of the control of *T. gondii* infection is carried out at home, especially in people that are most vulnerable to the parasite, such as pregnant women and immune-compromised persons. Primary prevention consisting of dietary recommendations, pet care measures, environmental measures, knowledge of risk factors and ways to control toxoplasmosis infection, has been found to be effective in reducing congenital toxoplasmosis [68].

At farm level preventive measures mostly apply to the pig industry where the parasite can be virtually eliminated by a set of hygienic measures, as discussed above. The serological prevalence of *T. gondii* in the pig population may be a useful indicator of the risk of human toxoplasmosis associated with the consumption of pork products. In the EU, the Commission Regulation No. 219/2014 modernised some specific requirements of the post-mortem inspection of pigs, favouring visual inspection instead of palpation and incisions [69]. However, it does not solve the *Toxoplasma* problem at slaughterhouse level. Therefore, the categorisation of risk for different types of farms (intensive systems and organic farms) would help the official veterinarian during ante-mortem and post-mortem visits [70].

Post-slaughter methods involve meat processing for human consumption and include mainly heat inactivation and freezing that are effective ways to kill the parasites if properly applied. Rani and Pradhan [71] studied the survival of *T. gondii* during cooking and low temperature storage and concluded that viable parasites were not found when the internal temperature of meat reached 64 °C and below −18 °C. Other meat curing procedures such as salting, smoking, or fermentation are less reliable for killing the parasites in meat because they are very much dependent of the concentration of the salt, the storage temperature, and the time of the processing [55]. The modern trend steered toward meat production from organic breeding, consumed raw or undercooked, with low concentrations of salt and additives (e.g., nitrites), may result in an increase of the zoonotic risk [54,56]. The current scientific knowledge is outdated and not sufficient for a full risk assessment. For this reason, innovative studies on *T. gondii* inactivation focusing on modern processing technologies may contribute to outline new preventive measures for consumers [55].

Currently, testing for parasite contamination in fresh produce is neither regulated nor mandatory. The increased popularity of consumption of raw and ready-to-eat vegetables may pose a new potential risk for consumers who could be accidentally exposed to oocysts, since most post-harvest processing measures do not guarantee the complete removal of oocysts or their effective inactivation [58].

Similar to other pathogens research is ongoing on the development of vaccines against toxoplasmosis. Currently, in animals, the only available vaccine is a live, attenuated, *T. gondii* S48 strain licensed for use in sheep in Europe and New Zealand for prevention of abortion [72]. However, using a live vaccine raises safety concerns for use in food-producing animals since the vaccine strain may revert to a wild type that might cause tissue cyst formation. DNA vaccination has been shown to determine long-lived humoral and cellular immune responses in vivo in animals [73]. Vaccination of cats has been proposed as the ultimate preventive measure because of the pivotal role of cats in the lifecycle of *T. gondii.* However, in case an effective vaccine for cats would be available, prospects on preventing oocyst-originated human toxoplasmosis by vaccination in large populations of cats are not favourable due to the large vaccination coverage needed [74].

### 2.4. Fasciola spp.

#### 2.4.1. Introduction

The trematodes *Fasciola hepatica* and *Fasciola gigantica* are the causative agents of the disease fasciolosis. The life cycle of *Fasciola* spp. includes plant-eating mammals (mainly ruminants, but also pigs and others) as final hosts, aquatic, lymnaeid snails as intermediate hosts and aquatic plants as carriers. Humans can also act as final hosts, and may even contribute to the perpetuation of the life cycle in endemic areas, where poor sanitation occurs [75], although the extent to which this actually occurs has never been quantified.

For decades, fasciolosis was perceived to be a purely veterinary problem [76], however, it is now also seen as an important disease in humans. As a response, WHO has listed fasciolosis as one of the neglected tropical diseases to be prioritized for control [77]. Human fasciolosis is known to occur worldwide, although it mainly affects the poorest communities in rural areas across subtropical and tropical countries. No recent burden data are available for fasciolosis. In 2012, Fürst et al. [78] estimated the global burden of fasciolosis at 35,206 DALYs, whereas the 2015 Global Burden of Disease estimate amounted to 90,041 DALYs, however the latter estimate came with a wide uncertainty interval (58,050–209,097) [79]. Estimates for the number of people infected range between 2.4 and 17 million [78,80,81], whereas those for the population at risk range between 91 and 180 million people [82,83]. Based on reported infection intensities, it has been estimated that 14% of fasciolosis infections are symptomatic [78]. These numbers could underestimate the true occurrence and impact of the disease, as for many regions, such as for instance a number of countries on the African continent, few or no community-based epidemiological surveys have been performed [84], and considering that the current burden estimation does not yet account for account for the immunosuppression, neurological or ocular effects due to fasciolosis.

Overall, fasciolosis is considered an emerging disease [82,85,86]. Its emergence could be due to the increased attention it received since its designation as neglected tropical disease, combined with factors facilitating its expansion and importance. For instance, climate change is thought to cause an increased risk for its snail host survival and distribution, and thus spread of the disease [87,88]. Moreover, the increased consumption of raw vegetables and fruits as part of increasingly popular healthy life styles and the introduction of sylvatic reservoir animals can assist in the spread of the disease [89,90].

Infected individuals harbour the adult *Fasciola* spp. worms, and unlike in *Clonorchis*/*Opisthorchis* spp. infection, will shed unembryonated eggs with their faeces/stool [91]. Once in a favourable freshwater environment, the eggs will then embryonate after approximately two weeks. After hatching, the miracidia will actively search for a suitable freshwater snail to penetrate and infect. In the snail, the miracidia will undergo several developmental stages (i.e., sporocyst and redia stages) and various rounds of asexual multiplication [90]. Next, an exponential number of free-swimming cercariae will leave the snail and encyst into metacercariae (MC) on plants growing in the same aquatic environment. Susceptible final hosts can acquire the infection by ingesting contaminated raw water plants. Upon ingestion, the MC will excyst in the small intestine, and penetrate the intestinal wall. Next, the juvenile flukes will migrate through the liver where they will mature in the bile ducts and start producing eggs, completing the life cycle [92]. Ectopic infections can also occur, with the juvenile or immature flukes erroneously migrating to subcutaneous tissues, gastrointestinal tract, heart, lung, or rarely, brain and eye [93,94].

In humans, fasciolosis can cause fever, abdominal colic, digestive disorders, weight loss, anaemia and jaundice due to fluke migration and subsequent destruction of the liver tissue, inflammation and blockage of the bile ducts [94]. In ectopic infections, symptoms and signs are specific to the organ affected by the migration path and presence of the flukes. Recently, it has been shown that neurological, meningeal and ocular symptoms, such limb and facial paralysis, speech disorders, blindness can also occur in hepatic fasciolosis, due to leakages in blood-brain barrier [95]. Finally, for a long time, *Fasciola* spp. infection was thought to cause parasitic pharyngitis, called Halzoun. The proposed route for acquiring this condition was the ingestion of raw liver, contaminated with immature or young flukes, attaching to the pharyngeal mucosa and invoking pain and bleeding. However, it is now argued the condition is rather due to infection with other parasites, such as *Linguatula serrata*, and *Dicrocoelium dendriticum* [90].

#### 2.4.2. Localisation of the Infection Risks for the Consumer in the Food Chain

Based on current knowledge, the most common pathway of infection for humans is the consumption of contaminated water plants. The best known infection source is watercress (*Nasturtium* spp.), an ubiquitous green leafy vegetable, with case reports around the globe mentioning the consumption of this plant in the anamnesis [96,97,98]. Furthermore, a wide range of other freshwater plants have been reported as infection sources, both wild and cultivated ones, such as dandelion (*Taraxacum* spp.) leaves, lamb’s lettuce (*Valerianella locusta*), and water spinach (*Ipomoea aquatica*). Contamination of freshwater plants is mostly due to the direct shedding of eggs by an infected host, being domestic livestock, humans or even sylvatic hosts, such as the nutria (*Myocastor coypus*), into an aquatic environment where the suitable intermediate snail host is present as well as the water plant eventually serving as carrier for the infective stage, metacercariae [90].

Next to water plants, a variety of terrestrial plants can carry the infective stage of *Fasciola* spp. For instance, infections due the consumption of lettuce (*Lactuca sativa*), and parsley (*Petroselinum sativum*) have been reported [99,100], while in other cases, infection was presumed to be due to consumption of wild aromatic plants, such as mint (*Mentha* spp.) [101]. Contamination of these plants can be due to them being submerged in water during a certain period of time, either in an environment where eggs had been shed, and thus snails were infected, or due to flooding of an adjacent field, with the runoff transporting infected snails or MC over a certain distance [102]. Other contamination pathways in terrestrial plants are washing in contaminated water bodies, and through irrigation with contaminated water.

Next to the ingestion of plants, the chewing and sucking contaminated plants are common infection routes for *Fasciola* spp. Well known examples are grass chewing, especially in children [103], as well as chewing on leaves of khat (*Catha edulis*), a popular tradition in the Horn of Africa and the Arabian Peninsula. Moreover, in certain regions, drinking beverages, such as herbal teas, and juices made from local plants are a known risk factor [104]. Drinking contaminated water is another suggested route of infection, although its importance is not well understood. Indeed, even though it is proven that unattached MC sink quite quickly to the bottom of water bodies, drinking contaminated water has been identified as the sole infection routes in a number of areas [105,106,107]. Naturally, ingestion of dishes and soups made with contaminated water, and washing of vegetables, fruits, tubercles, kitchen utensils or other objects with contaminated water can equally cause infection. Finally, in pig experiments, it was shown that the ingestion of raw liver with juvenile *Fasciola* spp. can lead to established liver infections [108], however to what extent this occurs in humans is not known.

#### 2.4.3. Diagnostic Options in the Food Chain

Surprisingly little attention has been given to the development of diagnostic tools for MC detection on plants and in water. Moreover, the sensitivity and specificity of available techniques have never been assessed. Up to now, most techniques for plants entail the screening of plant surfaces by means of a stereomicroscope [109]. Not only is this is not a feasible option in a commercial context, due to the time consuming nature of the method, it also requires the necessary expertise, to recognize *Fasciola* spp. MC, especially on thicker leave and stem surfaces, and to differentiate them from other digenean MC (e.g., *Paramphistomum* spp.) [109]. In water, while originally developed to investigate the contamination of rice fields, floats or buoys could be used to catch MC present in important waterflows from irrigation channels and/or streams [110,111]. Overall, cost-effective techniques to detect metacercarial contamination of consumed plants and water are lacking.

#### 2.4.4. Prevention and Control Options in the Food Chain

The most obvious method for fasciolosis prevention is avoiding oral contact with raw plants, whether it is the sucking/chewing or consumption of raw plants, especially those growing in an aquatic environment. However, culinary habits are often difficult to break, and therefore such recommendation might not be realistic for many communities and individuals. Another option would be the removal and/or destruction of MC attached on plant leaves. Unfortunately, washing vegetables using running water alone has proven to be only moderately successful in detaching MC [112]. On the contrary, briefly (5–10 min) soaking plants in vinegar (e.g., 120 mL/L) or liquid soap (e.g., 12 mL/L) solutions, seems effective in detaching and killing the MC [112,113], however these studies should be repeated to confirm the application of these methods in a commercial context. Some studies have investigated the effects of other chemical agents, of which potassium permanganate, and sodium hydroxide treatments seemed successful detachment/destruction methods [101,112,113], however, such agents have a considerable impact on plant palatability and appearance, and therefore their application seems unfeasible in a commercial context [113]. Cooking vegetables seems a more effective method to kill MC, however culinary traditions in raw plant consumption and in some regions, inadequate means to ensure sufficiently high temperatures while cooking, might hamper the application of this preventive measures. Similarly, boiling potentially contaminated water might not be feasible due to lack of means, in such regions filtration with an appropriate mesh size might be more effective [90].

Currently there are few preventive measures for fasciolosis implemented by governments. In the European Union, all bovines and small ruminants processed through abattoirs will be inspected for *Fasciola* spp., by means of visual inspection and incisions of the liver, in line with the European meat inspection legislation (EC 2019/627, [20]), however this inspection will not stop infected livestock from contaminating fields and therefore indirectly causing human fasciolosis cases. In a wider preventive context, plants commonly consumed by humans, should be grown under controlled conditions, inaccessible to snails, ruminants and other animals. Appropriate legislation has been implemented by a number of countries, e.g., France and Australia [90,114]. Moreover, the risk of contamination of these fields, due to run-off or irrigation with water from adjacent areas where *Fasciola* spp. can be present, should be excluded. A One Health approach is needed to ensure that all stakeholders are involved and informed about the disease, the risks and responsibilities. In any given region, appropriate fasciolosis prevention and control may only be achieved once the local transmission dynamics of *Fasciola* spp. are fully understood.

## 3. Pond/Ocean/River to Fork: Fish-Borne Parasites

In this chapter, three important fish-borne zoonotic parasites will be discussed, including *Clonorchis* spp. and *Opisthorchis* spp., related to freshwater fish, as well as the group of Anisakidae, related to marine fish (Figure 1).

### 3.1. Fish Borne Trematodes: Clonorchis, Opisthorchis spp.

#### 3.1.1. Introduction

Liver flukes of the Opisthorchiidae family, of which *Opisthorchis viverrini*, *O. felineus* and *Clonorchis sinensis* are the most important, cause opisthorchiasis and clonorchiasis in humans. The parasites have a complex lifecycle, requiring *Bithynia* spp. freshwater snails and cyprinid fish as primary and secondary intermediate hosts, respectively, and a fish-consuming mammal, such as humans, cats and dogs, as the final hosts [115]. Although cat and dogs are known to contribute to the life cycle of for instance *O. viverrini*, it has equally been shown for the same parasite, cats and dogs cannot sustain transmission in absence of humans as a final host [116]. It is not known, if the same is true for *O. felineus* and *C. sinensis*. The infected final host will discharge embryonated eggs in the biliary ducts and shed them via the stool/faeces. If shed in a suitable environment, the eggs will be ingested by the snail intermediate host. In the snail, the eggs will release miracidia that will subsequently develop into sporocysts, rediae and cercariae [117]. The latter developmental stage will be released from the snail host into the aquatic environment, where it will actively seek for a suitable secondary intermediate host, a freshwater cyprinid fish, penetrate its flesh or skin and encyst as metacercaria (MC) [118]. The final host acquires the infection by ingesting the raw or undercooked contaminated fish. Upon ingestion, the MC excysts in the small intestine and travels to the biliary tract via the ampulla of Vater. In the biliary ducts, the flukes will mature and start producing eggs, thereby restarting the life cycle [119].

Opistorchiosis and clonorchiosis can cause cholangitis, jaundice, cholecystitis, hepatomegaly and cholelithiasis [117]. Moreover, the International Agency for Research on Cancer has classified both *O. viverrini* and *C. sinensis* as Type I carcinogens. Indeed, in chronic infections, the (i) repeated mechanical damage due to the feeding and migrating flukes, combined with (ii) the secretion and excretion of metabolic products by the flukes as well as by *Helicobacter* spp. often co-infecting the final hosts, and (iii) the immunopathological response by the host, causing fibrosis and blockage of the bile ducts, may over time lead to oxidative damage to the epithelial cell DNA. Normal repair mechanisms and apoptosis are inhibited by certain fluke excretory/secretory products, and oncogenic mutations can occur as a consequence [117,120,121]. Eventually, the malignant transformations will lead to the development of cholangiocarcinoma (CCA), a highly lethal cancer of the bile duct, with an estimated median survival time of 4.3 months after diagnosis [121,122]. Up to now, while *O. felineus* has not been listed officially as a Type I carcinogen, there are indications both from animal experiments and reviews of the occurrence of cholangiocarcinoma in regions where the parasite is prevalent, that this fluke too has carcinogenic potential [123,124]. Opistorchiosis and clonorchiosis are two neglected yet emerging zoonotic diseases [82,125]. In the 1990s the total number of clonorchiosis and opistorchiosis cases was estimated at 7 and 10 million, respectively [83]. Nowadays, the total global number of infected people is estimated at about 20 million for *C. sinensis*, and at 10 million for *O. viverinni*, with most infections occurring in East Asia. Between 1.2 and 1.6 million people; mainly in Eastern Europe, are estimated to be infected with *O. felineus* [126]. Another 601 million people are thought to be at risk for *C. sinensis* infection, while 80 million for *Opisthorchis* spp. infection. The estimates for global burden due to clonorchiosis ranges between 275,370 and 522,863 Disability Adjusted Life Years (DALYS), whereas the estimates for opistorchiosis burden vary between 74,367 and 188,346 [78,79]. Based on reported infection intensities, it has been estimated that 8·2% of clonorchiosis, and 4·9% of opistorchiosis cases are symptomatic [78]. Increased importation of potentially contaminated fish, and newly acquired raw-fish eating habits might cause a future expansion in the distribution of the diseases [26].

#### 3.1.2. Localisation of the Infection Risks for the Consumer in the Food Chain

Humans principally acquire opistorchiosis and clonorchiosis through the consumption of undercooked or raw infected freshwater cyprinid fish [91]. Moreover, other preparation styles, such as fermentation, pickling, inadequately freezing, or smoking of fish can pose a risk to the consumer [127]. In addition to cyprinid fish, some other fish types such eleotrids, cichlids, and osmerids are known to harbour MC [128]. Finally, some freshwater shrimp species have been reported to carry MC, and their consumption could thus pose the consumer at risk for infections [128].

While it is clear that the MC only encyst in fish/shrimp tissue, and thus the consumption of these tissues is the main infection source for acquiring the diseases, it is not well understood whether the preparation of contaminated fish can lead to contamination of the cooking environment, and thus subsequent human infection via contact with this environment. Indeed, *Opisthorchis* and *Clonorchis* spp. MC are distributed over different parts of the fish body, being the muscles, fins, heads and organs [129]. Therefore, it is not unimaginable that cutting boards might become contaminated with MC during the preparation of the fish dish. For instance, in their study performed in Laos, Araki et al. [130] reported on the observation that household cooks clean their chopping boards by scratching them using a knife and running water, and at times fish scales could still be found on the chopping boards after cleaning. Likewise, utensils and hands might become contaminated during food preparation or while eating [130]. Such contamination could explain infections in people reporting to never consume raw fish [131]. Finally, it has been hypothesized that people could become infected by drinking water contaminated by MC released from dead fish tissues, although this has never been proven to occur under field conditions [128].

#### 3.1.3. Diagnostic Options in the Food Chain

All techniques to detect *Opisthorchis* spp. and *C. sinensis* MC in fish are based on postmortem investigations. Most consist of rather labour-intensive, traditional parasitological techniques. The most basic method is the direct compression method: fins, muscle, scales and subcutaneous tissues are collected, compressed between glass slides and examined for MC under a stereomicroscope [83]. In a second commonly used method, the artificial digestion method, the fish is divided in five parts (head, anterior and posterior trunk, tail and subcutaneous tissue). The tissues are subsequently ground, then digested using an artificial gastric juice (mostly a pepsin-HCl solution), and after sedimentation, MC are sought using a stereomicroscope [83]. A systematic review has shown that studies applying the compression method find higher prevalence estimates than those using the digestion method, however, their performance has not been compared directly [132]. Either way, both techniques require expert knowledge on the morphology of the different MC present in fish to allow for their correct differentiation (e.g., of Opisthorchiidae, Heterophyidae and Lecithodendriidae) [133].

Molecular techniques are however available to ensure correct differentiation of MC. For instance, a multiplex PCR was developed, targeting mitochondrial DNA, to allow differentiation of *Clonorchis* and *Opisthorchis* MC, particularly useful for regions where their distribution overlaps [134]. Moreover, a loop-mediated isothermal amplification (LAMP) was also developed for *C. sinensis* detection in fish, with a markedly higher sensitivity as compared to PCR [135]. Nevertheless, such molecular techniques remain expensive and not available in a commercial aquaculture context. Up to now, quick, easy, cheap diagnostic tools with adequate test performance are still lacking for *Clonorchis* and *Opisthorchis* detection in fish.

#### 3.1.4. Prevention and Control Options in the Food Chain

Prevention of clonorchiosis and opisthorchis entails preventing the consumption of raw or undercooked contaminated fish/shrimps and avoiding contact with a contaminated environment. In many communities, the consumption of raw or undercooked fish is however deeply engrained in the culinary and social culture. For instance, the consumption of raw fish is often part of a social drinking events for males in many Asian countries [136,137,138]. Simply halting its consumption or changing the preparation style might therefore not be feasible. Moreover, MC are moderately tolerant to several preparation and preservation styles, which would usually be considered as effective in killing off germs. For instance, freezing at −12 °C had to be continued for 480 h to inactivate *Clonorchis* MC, whereas cooking at 50 °C required five hours to inactivate unspecified MC [127]. The only effective method is thoroughly heating the fish at a high temperature (e.g., 65 °C for 1 min) [127]. Another route for prevention in the intermediate host, namely a vaccine for freshwater fish, is currently being explored. Indeed, an oral vaccine based on *Bacillus subtilis* expressing enolase, was developed and is being tested [139,140]. In a wider preventive perspective, the life cycle can be broken by disconnecting human and animal faeces from the aquatic environment. At the moment, in some countries, the use of toilet types draining stool directly to ponds, still persist [141], and animal and human faeces continue being used as fish feed [139], therefore the transmission perpetuates. In Thailand, an opistorchiosis control program, called the “Lawa model” was developed. This EcoHealth/One Health-inspired approach combining human treatment with novel community-based and school health education, ecosystem monitoring and community participation, was implemented in the opistorchiosis endemic area at Lawa Lake, Khon Kaen province, Thailand [142]. The program successfully cut back infection rates in both humans, fish and snails, and will now be scaled up to other regions in Thailand and beyond [143].

### 3.2. Anisakidae

#### 3.2.1. Introduction

Nematodes from the family Anisakidae are by far the most prevalent macroparasites in fish implicated in human disease. In their adult stage, anisakids are mostly found in the stomach of marine mammals as their definitive hosts, whereas the larval stages are found in smaller invertebrates, such as crustaceans as their first intermediate host, and in fish as their second intermediate host. Many commonly exploited marine fish species are infected, with the main zoonotic anisakid species being, although not limited to these, *Anisakis simplex* sensu stricto, *A. pegreffii*, *Pseudoterranova decipiens*, and *Contracaecum osculatum* [144]. Data on their prevalence in fish are known for many fish species with varying prevalences (e.g., up to 100% in herring) dependent on the geographical fishing grounds and seasons [145,146]. Yet ultimately, almost all species of teleost fish throughout the oceans may act as hosts where larvae can be found in the gastrointestinal tract or musculature of the fish [147,148].

Human infection, collectively named anisakidosis, takes place after consumption of undercooked fish containing a viable third-stage larva (L3), and may lead to several gastro-intestinal symptoms depending on the localisation of the larvae (acute, chronic, and ectopic) [149]. In addition to abdominal symptoms, a series of thermostable allergens present in *A. simplex* and *A. pegreffii* may also compromise human health with acute allergic manifestations ranging from urticaria, angioedema, asthma, conjunctivitis, to even a potential lethal anaphylactic shock [150]. The number of human health problems related to Anisakidae has not been fully quantified as global awareness of this foodborne parasite is still in its upsurge. Collection of more epidemiological data on the disease is therefore encouraged to provide better insights into the impact of Anisakidae on human disease. Nevertheless, specific country case studies from Spain and Norway have estimated 10,383–20,978 annual anisakidosis cases, as well as a 22% prevalence value of *Anisakis*-sensitization in certain regions [151,152]. Furthermore, it was stated that the most frequent cause of an anaphylactic episode due to a hidden allergen, is fish infected with *A. simplex* [153]. Finally, sufficient proof-of-principle has been provided in the past that demonstrates the transmissibility of anisakid allergenic peptides from fishmeal, as a feed component, to aquacultured fish and chicken meat [154,155,156,157]. These findings may significantly change the importance of these zoonotic nematodes from originally a purely fish borne food risk, to a much wider risk from several food sources (pork meat, chicken meat, aquacultured fish, etc.).

#### 3.2.2. Localisation of the Infection Risks for the Consumer in the Food Chain

As abovementioned, consumers obtain an anisakid infection via consumption of infected fish that has not been sufficiently frozen or cooked [149]. Different preventive measurements can be taken to avoid this (see below), though these are primarily related to the infection risk with viable larvae. By adequate cooking or freezing, *Anisakis*-sensitized consumers remain at risk given the thermoresistant characteristics of the allergens [158]. Moreover, removal of the larva does not at all guarantee freedom of allergens since some of these anisakid allergens are excretory-secretory products excreted/secreted by the larval body during its migration through the fish flesh. As such, patients may be exposed to them when consuming fish, even though the larvae might have been removed during the quality control of the fish [159]. Finally, and different to other food allergies, occupational allergies in aquaculture and fishery workers after inhalation and/or skin contact with anisakid allergens have been reported [160].

#### 3.2.3. Diagnostic Options in the Food Chain

Fish and fishery products can be examined for the presence of anisakid larvae and traces by a variety of detection methods. In the industrial setting (i.e., on the boat, in processing plants), candling is the most routinely used method for the detection of anisakids in commercial fish fillets. It entails a brief visual inspection of fish fillets on a light table to spot and manually remove parasites [161]. While this technique has the major advantage of not affecting the fish quality and thus allowing consumption afterwards, it is labour intensive and as such highly costly. Moreover, studies report a poor sensitivity with up to 76% of the larvae not being recovered, although this is dependent on the fish (colour, thickness, skin), the larvae (size, colour), and the skills of the inspector [161,162,163,164]. In laboratory settings on the other hand, a range of highly accurate alternatives such as UV press method, enzymatic digestion, and immunoassays are available and implemented, though the complete destruction of the fish tissue, renders these methods unfit for the application in the industry [165,166,167]. As a result, candling is still the standard method for the detection and removal of anisakids from fish fillets on an industrial scale. Future research should thus look into the development of more accurate, fast, non-destructive scanning methods to replace candling.

As dead larvae may still be responsible for allergic reactions in sensitized consumers, other tools directly targeting anisakid proteins have been developed [168]. However, the presence of anisakid proteins does not necessarily correlate with an allergic reaction since not all proteins are allergens. To deal with this, liquid chromatography tandem mass spectrometry methods and allergen specific enzyme-linked immunosorbent assays are available [169,170], but high equipment costs and their destructive entity still hamper their use in an industrial setting.

#### 3.2.4. Prevention and Control Options in the Food Chain

Prevention of gastro-intestinal anisakidosis is simply based on avoiding the ingestion of a live L3 in raw/undercooked fish. In the food chain, this can be accomplished by maintaining a cold chain from boat to plate and by immediate gutting of the fish on the boat in order to avoid *post-mortem* migration from the fish gut to musculature. Care must be taken to destroy these viscera rather than disposing them in the sea water as this practice results in, once again, dissemination of the parasites [171]. Freezing of the fish has also been recommended by public health agencies and included in the current legislation of the European Union and Japan [172,173]. Specifically, food industry business that sell fish intended for raw, marinated, or salted consumption, must first freeze the fish in its entirety at −20 °C for >24 h, or −35 °C for >15 h to ensure killing of the larvae [174]. It is to be expected though, that in a big container of fish, not all parts of the fish will reach a temperature to kill all larvae. Ideally, freezing should therefore be followed by a period of storage in the frozen state to ensure complete elimination of the parasite. In addition to freezing/cooking, a visual check for larvae by the fish industry can be conducted by ways of candling (see above). The abovementioned limitations of this tool, however, make this tool insufficient for full clearance of the parasite and emphasizes the importance of adequate deep-freezing. Finally, at the stage of not only the consumer, but also the relevant governmental institutes and medical/veterinary staff, raising awareness regarding the presence of these parasites and possible preventive measures (e.g., cooking, consumption behaviour) is a principle preventive measure that should be taken.

While a variety of measures can be taken to reduce the incidence of human gastro-intestinal anisakidosis, it is important to consider that an *Anisakis*-sensitized individual may still develop an allergic reaction if the larva is dead/removed or if its traces are present [158]. So far, no allergen destruction process has been discovered, and standard testing for anisakid allergens is up to date not conducted on any food type. A suggestion, however, could be to label high-risk products for the possible presence of *Anisakis* allergens to warn sensitized patients who do not tolerate even properly cooked or canned fish.

## 4. Discussion and Conclusions

In this paper we have attempted to highlight the presence of a number of FBPs in different food chains, the risk of infection of humans by parasites, and their diagnostic and control challenges. We have also highlighted the complexity of their life cycles whereby different parasite stages, infective to the human host, may occur at different places within a food chain, for example, in the case of *T. solium* whereby the meat-borne component will place humans at risk when consuming pig carcasses infected with viable cysticerci, but humans may also be at risk when consuming vegetables or fruits contaminated with the parasite’s eggs from the farm, or via contaminated water. *Toxoplasma gondii* is also a good example of multiple complex ways of transmission to humans.

As mentioned, the increased popularity of consumption of raw and ready-to-eat vegetables may pose a new potential risk for consumers, since most post-harvest processing measures do not guarantee the complete removal of oocysts/eggs or their effective inactivation [58]. The same can be said for raw fish/meat consumption, considering the generally low sensitivities or simply non implementation of detection tools.

We have also highlighted how interactions in interface areas may lead to the introduction of parasites from the *forest to fork* food chain into the *farm to fork* food chain. The impact of increasing contact between wildlife, livestock and humans on food safety needs to be considered carefully, especially bearing in mind the trend towards outdoor farming [3] and the increase in relevant wildlife species such as the wild boar [27].

The multidisciplinary One Health approach will be needed to deal with the impact of globalisation and climate change on the transmission of foodborne parasites to humans [26]. The One Health approach is increasingly applied by governments, yet mostly considering human and animal components. The environment is the most understudied component of parasite transmission, yet is very important. There is an urgent need for researchers to take up this work, e.g., to develop highly performing diagnostic tools to detect environmental stages, to assess their viability, etc.

The availability of cheap, easy to use, highly performing diagnostic tools fit for purpose is another challenge. While an increased amount of effort has been put into developments of new tests in different formats, and target product profiles are being developed (e.g., for *T. solium* [175]), their large-scale evaluation in relevant matrices is often lacking. Integrated systems looking into more integrated sample collections where relevant (e.g., environmental samples, meat samples from pig farms with outdoor access), and test systems allowing multipathogen detection are other aspects that need to be considered.

Monitoring and surveillance, and improved reporting based on proper diagnostics would be useful to implement for a number of FBPs. As resources are always limited, risk-based surveillance might help in the prioritisation, and lead to an efficient and effective allocation of, resources [21]. For *Trichinella* spp. and *T. saginata* this system has been put in place in the EU (see Section 2.1.4 and Section 2.2.4).

Integration may be considered not only on a detection level, but also on a prevention/control level, where chosen interventions may impact on several pathogens. The implementation of highly controlled and confined farming systems has led to commercial pork originating from these farms to be free of *Trichinella* spp. These high levels of biosecurity not only impact the presence of *Trichinella* spp., but also *Taenia* spp. and *T. gondii* are rarely detected. Moreover, a focus on herd health and management, biosecurity at the farm level would tackle not only a number of FBPs, but also other pathogens such as Salmonella and Campylobacter, and this at a primary, pre-harvest level [176]. Nevertheless, the trend towards farming systems with more outdoor access, encouraged by animal welfare expectations, increase infection risk [3]. The latter should be compensated with either pre-harvest (e.g., improved detection) or post-harvest (e.g., inactivation via cooking) measures.

To finalise, the consumer also plays an essential role in risk of exposure, via human behaviour in choice of food to consume but also in the culinary practices in how to process food for consumption. While cooking at a sufficient temperature would deal with all parasites described above, often, this is not done. Other practices such as marinating, salting, drying, smoking are more often than not, insufficient to inactivate the parasite. From a different perspective, human migration and import of infected animals from endemic to non-endemic areas may lead to a (re)introduction of pathogens [3]. Consumer education regarding the risks and awareness creation would be highly beneficial.
